# Experimental evidence that ecological effects of an invasive fish are reduced at high densities

**DOI:** 10.1007/s00442-014-2899-5

**Published:** 2014-02-17

**Authors:** Matthew S. Kornis, Jedchada Carlson, Gabrielle Lehrer-Brey, M. Jake Vander Zanden

**Affiliations:** 1 Center for Limnology, University of Wisconsin-Madison, 680 N. Park Street, Madison, WI 53706 USA; 2 Present Address: Smithsonian Environmental Research Center, 647 Contees Wharf Road, Edgewater, MD 21037 USA

**Keywords:** Species interactions, Invasive species, Round goby, Density impact relationship, Great Lakes

## Abstract

Understanding the relationship between invasive species density and ecological impact is a pressing topic in ecology, with implications for environmental management and policy. Although it is widely assumed that invasive species impact will increase with density, theory suggests interspecific competition may diminish at high densities due to increased intraspecific interactions. To test this theory, we experimentally examined intra- and interspecific interactions between a globally invasive fish, round goby (*Neogobius melanostomus*), and three native species at different round goby densities in a tributary of the Laurentian Great Lakes. Eighteen 2.25 m^2^ enclosures were stocked with native fish species at natural abundances, while round gobies were stocked at three different densities: 0 m^−2^, 2.7 m^−2^, and 10.7 m^−2^. After 52 days, native fish growth rate was significantly reduced in the low density goby treatment, while growth in the high density goby treatment mirrored the goby-free treatment for two of three native species. Invertebrate density and gut content weight of native fishes did not differ among treatments. Conversely, gut content weight and growth of round gobies were lower in the high goby density treatment, suggesting interactions between round gobies and native fishes are mediated by interference competition amongst gobies. Our experiment provides evidence that invasive species effects may diminish at high densities, possibly due to increased intraspecific interactions. This is consistent with some ecological theory, and cautions against the assumption that invasive species at moderate densities have low impact.

**Electronic supplementary material:**

The online version of this article (doi:10.1007/s00442-014-2899-5) contains supplementary material, which is available to authorized users.

## Introduction

Species invasions have altered ecological communities worldwide, but impact can vary greatly among species and habitats (Williamson and Fitter 1996; Melbourne et al. 2007). Introductions often alter food webs through new interactions with indigenous species, making it difficult to predict invasion effects (Vander Zanden et al. 1999; Bruno et al. 2005). Species introductions are commonly associated with negative effects on native species through predation, or competition for food or habitat (Mack et al. 2000; Mills et al. 2004; Hermoso et al. 2011). They can also benefit native species by becoming an important prey source (King et al. 2006) or through habitat modification or competitive or predatory release (Rodriguez 2006).

Invasive species effects are generally assumed to increase with density, but density-impact relationships vary among species, particularly regarding the threshold density at which significant impacts emerge (Yokomizo et al. 2009). There is a pressing need to understand relationships between density and impact because invasive species abundance is spatially heterogeneous (Hansen et al. 2013) and because such relationships are poorly documented for even the most notorious invasive species (Kulhanek et al. 2011). In addition, intraspecific interactions frequently emerge at high densities, and can limit a species’ own population growth by negatively affecting recruitment (Ricker 1954), growth (Stiling 1988; Post et al. 1997), and resource availability (Denno and Roderick 1992), or by increased aggressive intraspecific interactions (Magnuson 1962). Invasive species are not immune from self-regulating effects: invasive species sometimes follow a “boom–bust” population trajectory, where high initial abundance stabilizes at lower densities over the long term (Simberloff and Gibbons 2004; Strayer and Malcom 2006). Therefore, intraspecific interactions amongst invasive species could potentially mediate their ecological effects.

Instances in which an invasive species’ impact derives from strong negative interactions with native species may provide insight into how intraspecific interactions might mediate density-impact relationships. There are numerous examples of invasive species exerting negative effects on native species by being a superior competitor (Vander Zanden et al. 1999; Bergstrom and Mensinger 2009; Cucherousset and Olden 2011). However, theory based on observations of species within their native range predicts that intraspecific interactions should be stronger than interspecific interactions at high densities (Connell 1983; Goldberg and Barton 1992; Mangla et al. 2011). This may be because resource requirements are more similar for conspecifics than heterospecifics (Goldberg and Barton 1992; Mangla et al. 2011), or because conspecifics are perceived as greater rivals than heterospecifics (Walls 1990; Macedonia and Stamps 2010). Therefore, negative interactions between invasive and native species may be reduced if intraspecific interactions are prevalent when an invasive species is at high density.

To test this hypothesis, we investigated interspecific and intraspecific interactions involving invasive round gobies *Neogobius melanostomus*, a fish with introduced populations in Eurasia, the Laurentian Great Lakes and their tributaries. Round gobies frequently affect native ecosystems through negative interactions with native biota, including benthic invertebrates (Barton et al. 2005; Lederer et al. 2008) and bottom-oriented fishes (Kornis et al. 2012). For example, the abundance of slimy sculpin *Cottus cognatu*s (Janssen and Jude 2001), Johnny darter *Etheostoma nigrum* (Lauer et al. 2004), and logperch *Percina caprodes* (Balshine et al. 2005) declined following introduction of round gobies in the Laurentian Great Lakes, as did the abundance of European flounder *Platichthys flesus* after round goby introduction in the Baltic Sea (Karlson et al. 2007). Round gobies dominate food resources in laboratory experiments with native fish species (Bergstrom and Mensinger 2009), and are also aggressive defenders of territory (Wicket and Corkum 1998). In nature, aggressive interactions with native species can lead to spawning interference and displacement of native fishes to suboptimal habitats (Dubs and Corkum 1996; Janssen and Jude 2001; Balshine et al. 2005). Round gobies also exhibit negative interactions with conspecifics, although less is known about their frequency in nature. Stammler and Corkum (2005) found that round gobies infrequently display aggression towards conspecifics in aquaria experiments. However, a similar experiment found that resident round gobies displayed aggression toward intruding gobies, and spent more time in refuge when intruding gobies were present (Fitzsimmons et al. 2006). Fitzsimmons et al. (2006) also found that tanks containing one, five, and ten round gobies consumed similar amounts of total food, suggesting that per capita food consumption declined with increased goby density. Consequently, round gobies are an excellent case species for investigating whether intraspecific interactions amongst invasive species can mediate ecological effects on native species. The objectives of this study were to (1) assess intra- and interspecific interactions between round gobies and native fishes by evaluating individual growth rates at three levels of round goby density, and (2) examine fish diets and benthic invertebrate density to determine the degree to which growth rate patterns could be attributed to competition for food.

## Materials and methods

### Site description

During the summer of 2009, 18 experimental enclosures were installed 10.3 km upstream from Lake Michigan in a 250 m long reach of the Little Suamico River, northeast Wisconsin, USA. This reach provided a homogenous environment that contained an abundance of round gobies and native fish species. Environmental data were collected on 14 September 2009 from 18 transects (one near each enclosure) taken from bank to bank under typical flow conditions. Flow velocity (Swoffer 2100 flow meter) and depth measurements were collected at three points along each transect at 25, 50, and 75 % of total stream width. Stream width ranged from 6.9 to 11.5 m (average 8.9 m), depth ranged from 0.2 to 0.45 m (average 0.29 m) while maximum flow velocity (amongst the three measurements) ranged from 0.02 to 0.15 m s^−1^ (average 0.07 m s^−1^). Substrate (visually assessed) was predominantly cobbles and boulders on top of sand and gravel. Although riffles and pools were present, run microhabitat predominated and all enclosures were placed in run habitat. Three short floods occurred during the experiment, but the stream returned to base flow within 48 h of each flood and water levels never exceeded the height of the experimental enclosures.

Round goby density in the experimental reach was estimated to be 0.43 m^−2^ from 3× depletion sampling in a blocked off reach. However, round gobies were not homogeneously distributed, and densities ranged from 2 to 4 m^−2^ in patches where gobies were captured. Present native fishes included common shiner *Luxilus cornutus*, creek chub *Semotilus atromaculatus*, hornyhead chub *Nocomis biguttatus*, Johnny darter, longnose dace *Rhinichthys cataractae*, and white sucker *Catostomus commersonii*.

### Experimental design

**Fig. 1:**
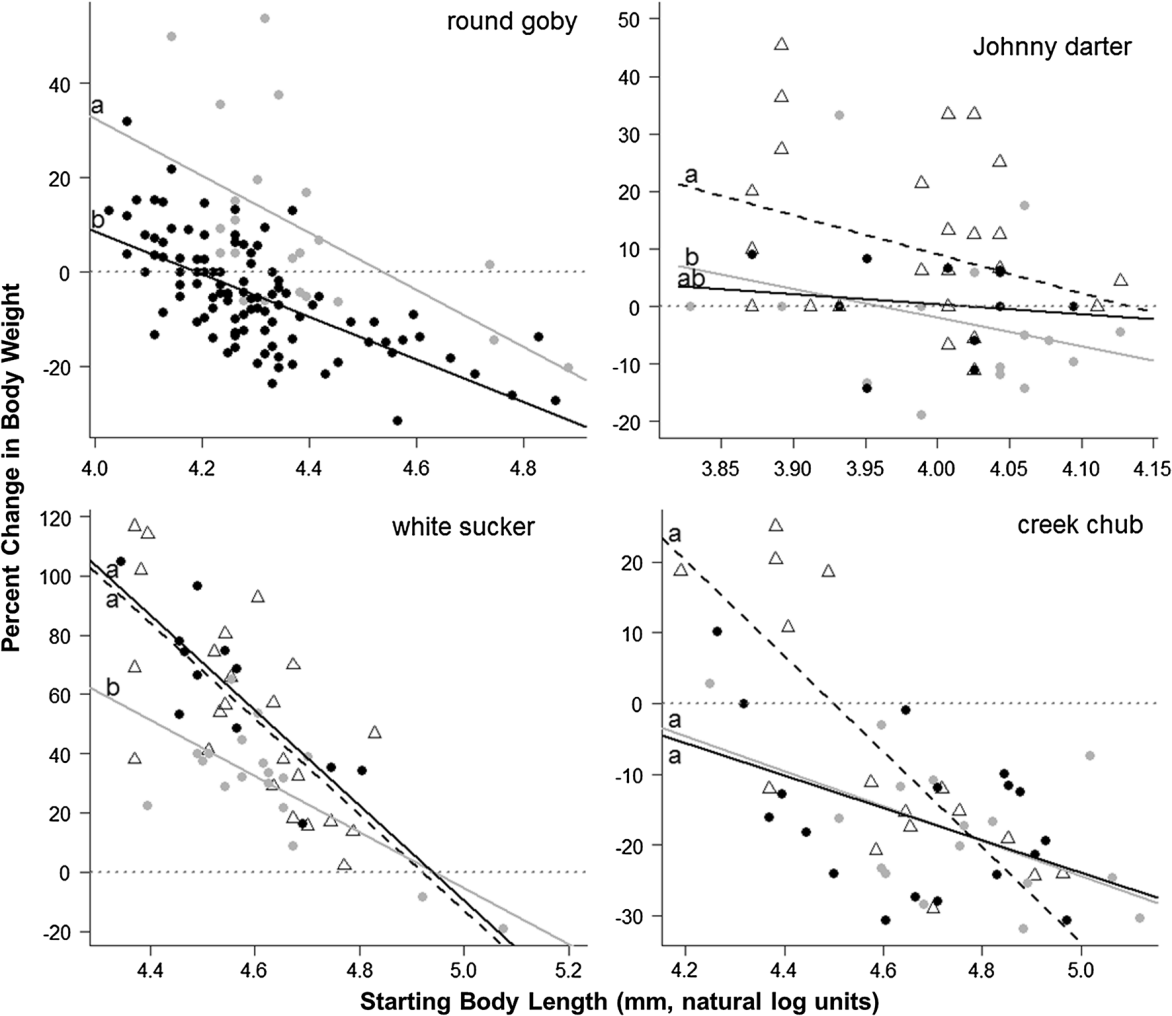
Treatment-related differences in relative growth rate of round goby (*Neogobius melanostomus*), Johnny darter (*Etheostoma nigrum*), white sucker (*Catostomus commersonii*) and creek chub (*Semotilus atromaculatus*). *Open triangles* and *dashed lines* are from the goby-free treatment (0 gobies m^−2^), *gray circles* and *solid gray lines* are from the low-goby treatment (2.7 gobies m^−2^), and *black circles* and *solid black lines* are from the high-goby treatment (10.7 gobies m^−2^). For all panels, relative growth is expressed as percent change in body weight from the start to the end of the experiment (7 July 2009 to 14 September 2009, Little Suamico River, Wisconsin, USA). Each *point* represents an individual fish. Treatments labeled *a* are significantly different from treatments labeled *b* at the *P* < 0.05 level, while treatments labeled *ab* are not statistically different from either *a* or *b* groups. Statistical differences amongst treatment levels were derived from post hoc pairwise comparisons following ANCOVAs with fish length as a covariate. Note that *y* axes are not consistent among panels; *horizontal dotted lines* in each panel represent a zero percent change in body weight. *n* = 5, 5, and 6 for number of replicates in the no goby, low goby, and high goby treatments, respectively

A complete description of enclosure construction, as well as photographs of the installation process, can be found in Appendix 1 of the Electronic Supplementary Material (ESM). Enclosures with closed bottoms measuring 1.5 × 1.5 × 0.6 m were installed on alternating banks of the stream at least 12 m apart within the same 250 m stream reach (GPS coordinates of upstream and downstream endpoints were 44.713304, −88.067453 and 44.712107, −88.069647). To simulate natural substrate conditions, fine substrate (sand and gravel) was placed inside the enclosures, followed by cobbles and boulders from an undisturbed section of stream to minimize invertebrate loss (ESM Fig. 1). Water temperature, depth, and flow velocity were measured within and adjacent to each enclosure on the same day as the environmental transects described above to compare enclosure conditions to the ambient environment. Enclosures were left for 7 days prior to the introduction of fish to allow recolonization by invertebrates. We deemed 7 days as sufficient to allow invertebrate biomass to resemble that of the ambient stream because the enclosures were set up using substrate from undisturbed areas of the stream that still had invertebrates attached (see ESM Appendix 1 and Fig. 1). Moreover, geometric mean dry biomass in the enclosures after the 7 day recolonization period was 1,420 g m^−2^, compared to 909 g m^−2^ in the enclosures (*T*_32_ = 1.5, *P* = 0.14) and 750 g m^−2^ in the ambient stream (*T*_19_ = 1.2, *P* = 0.26) at the end of the experiment. Therefore, there was no evidence that invertebrate biomass was depleted at the start of the experiment from enclosure installation effects.

Enclosures were randomly assigned into one of three treatment groups spanning a gradient of round goby density (0, 2.7, and 10.7 round gobies m^−2^), with six enclosures per treatment. Homogenous habitat throughout the experimental stream reach and within each enclosure enabled this completely randomized design. Throughout this paper, treatment levels are referred to as no goby, low goby, and high goby. These densities were chosen to reflect round goby densities observed in uninvaded streams (0), invaded streams (2.7), and areas of the Great Lakes (10.7). Round goby density often exceeds 10.7 m^−2^ in the Great Lakes, and has been occasionally observed at 100 m^−2^ (Corkum et al. 1998; Steinhart et al. 2004). Stream densities are less well known, and we selected 2.7 m^−2^ based on our observations from Lake Michigan tributaries. All treatments also contained creek chubs (four individuals, 1.8 m^−2^), white suckers (four individuals, 1.8 m^−2^), and Johnny darters (six individuals, 2.7 m^−2^), which shared a preference for the homogenous run habitat that typified the experimental reach. Three other species common in the experimental reach were excluded from enclosures due to tagging-related mortality (common shiner), preference for high velocity riffle microhabitat (longnose dace), and similarity to creek chubs (hornyhead chub). Fish were collected on 7 July 2009 using a battery powered DC backpack electro-fishing unit (ETS Electrofishing LLC, model ABP-3), and each individual was weighed, measured for length, and tagged with a visible implant elastomer tag to provide individual growth records. Fishes were collected from within the 250 m experimental reach, and from a 200 m reach immediately downstream of the experimental reach. Tagged fishes were retained in aerated containers for an hour prior to release in the enclosures to ensure recovery from the tagging procedure.

### Data collection at end of experiment

At the end of the 52-day study period (7 July 2009 to 14 September 2009) each enclosure was sampled twice for invertebrates using the Hess sampler described above; mean values from these subsamples were used in statistical analyses. Substrate was then removed to facilitate recapture of fish. Enclosures were electrofished every 5 min until three consecutive passes failed to produce any fish. Recaptured fish were identified by tag, measured for length and weight, humanely euthanized, and preserved in ethanol for laboratory diet analysis. Two enclosures (one each from the no goby and low goby treatments) were removed from the experiment due to poor recapture rates (20 and 28 % fish recaptured) and holes in the mesh.

### Laboratory analyses

Diet items from a subset of fish were extracted under a Leica MZ6 dissecting scope, identified to order (or classified as unidentifiable), and enumerated. Identification keys included Peckarsky et al. (1990) and Merritt and Cummins (1996). Stomach contents were dried for at least 48 h at 60 °C and weighed to the nearest 0.1 mg. Stomach contents were examined for 105 round gobies, 36 Johnny darters, 33 creek chubs, and 26 white suckers; a minimum of seven individuals per species were examined in each treatment. Diet overlap between round gobies, Johnny darters, and creek chubs was calculated using Schoener’s index, which expresses overlap on a scale from 0 (no overlap) to 1 (identical diets) (Schoener 1971). Diet overlap with white suckers was not determined because their diets were mostly unidentifiable.

Invertebrates were dyed with rose bengal, enumerated, and dried for at least 48 h at 60 °C. Members of Ephemeroptera, Trichoptera, and Coleoptera were identified to family level and Dipterans were classified as Chironomidae if applicable; all other invertebrates were classified to order level. Dried invertebrates were weighed to the nearest 0.1 mg.

### Statistical analyses

Individual growth rates can be used to evaluate interaction strength amongst and within species (Britton et al. 2011). In this study, relative growth rates of individual fish were expressed as a percentage of body mass at the start of the experiment:

$${\text{Relative growth rate = 100 }} \times \frac{\text{End weight} - {\text{Start weight}}}{\text{Start weight}}$$

Relative growth and total dry weight of stomach contents (hereafter “gut content weight”) were expected to covary with body size. To account for this, we analyzed treatment effects on fish growth and gut content weight using a single-factor analysis of covariance (ANCOVA), with treatment (no, low, and high goby) as the grouping factor and fish length as a covariate. ANCOVAs were interpreted using Type I sum of squares (SS), which is appropriate when testing for the additional effect of a predictor beyond the effects of a known covariate. In our case, Type I SS allowed for assessing the effects of round goby density after accounting for variation in percent growth explained by fish length. Significant ANCOVAs were followed by Tukey–Kramer post hoc comparisons (based on differences of least squared means) to identify which treatment groups were significantly different from one another. The interaction between treatment and fish length was also considered in all initial models. Significant interaction terms (*P* < 0.05) suggest treatment effects only on individuals of certain length. Therefore, additional ANCOVAs were conducted on small and large size classes of species with significant interaction terms.

Treatment effects on total invertebrate biomass (both pre- and post-experiment), taxon-specific invertebrate biomass, and fish recapture rates were analyzed using one-factor analysis of variance (ANOVA) followed by Tukey–Kramer HSD post hoc comparisons. Environmental conditions between enclosures and the adjacent ambient environment were compared using paired *t* tests. Fish length, invertebrate mass, and gut content weight were log_e_ transformed to meet the assumption of normality. ANCOVA analyses were performed using SAS (V.9.2, Proc GLM procedure); all other analyses were performed in R (R Development Core Team 2010, version 2.13.2).

## Results

### Environmental characteristics

There was minimal variation in water temperature (5.6 ± 0.2 °C), flow velocity (0.02 ± 0.01 m s^−1^) and average depth (0.30 ± 0.05 m) amongst enclosures (average ± standard deviation for all). Enclosures reduced flow relative to immediately adjacent areas (0.02 vs. 0.05 m s^−1^ on average, *T*_17_ = 3.67, *P* = 0.002). However, flow velocity was comparable to non-thalweg areas of the natural stream (0.02 vs. 0.02 m s^−1^ on average, *T*_17_ = 0.55, *P* = 0.59). Neither depth (0.30 vs. 0.29 m on average, *T*_17_ = 0.53, *P* = 0.60) nor temperature (5.6 vs. 5.6 °C on average, *T*_17_ = 0.44, *P* = 0.67) differed significantly between enclosures and adjacent stream habitat.

### Fish relative growth rates

Round goby density had a significant effect on the relative growth of round goby (*F*_1,120_ = 53.5, *P* < 0.001), Johnny darter (*F*_2,49_ = 4.1, *P* = 0.02), and white sucker (*F*_2,47_ = 6.3, *P* = 0.004) (Fig. 1) after accounting for significant negative correlations between length and relative growth rate for all species. The mean relative growth (mean ± S.E.) of round gobies was significantly greater at low densities (9.6 ± 4.2 %) than high densities (−4.7 ± 1.1 %) (*T*_120_ = 7.3, *P* < 0.0001). Johnny darter and white sucker relative growth rates were lowest in the low-goby treatment (Fig. 1). White sucker growth was significantly higher in the no-goby treatment (54.1 ± 6.8 %) and high-goby treatment (62.7 ± 7.5 %) than the low-goby treatment (30.0 ± 4.7 %) (no-goby vs. low-goby *T*_39_ = 3.2, *P* = 0.006; high-goby vs. low-goby *T*_28_ = 3.1, *P* = 0.01). Johnny darter growth was greater in the no-goby treatment (10.3 ± 3.1 %) than the low-goby treatment (–2.5 ± 3.1 %) (*T*_42_ = 2.7, *P* = 0.03), while growth in the high-goby treatment (0.5 ± 2.4 %) was not significantly different from either the low-goby (*T*_26_ = 0.5, *P* = 0.66) or no-goby (*T*_36_ = 1.8, *P* = 0.17) treatments.

For creek chubs, there was a significant interaction between treatment group and fish length (*F*_2,44_ = 4.5, *P* = 0.02) that reflected higher growth rates for smaller creek chubs in no-goby enclosures (Fig. 1). A model that only included creek chubs ≤110 mm (log length of 4.7) found a significant treatment effect (*F*_2,16_ = 5.28, *P* = 0.02) and no significant interaction (*F*_2,16_ = 1.4, *P* = 0.28). For creek chubs ≤110 mm, growth rate was significantly higher in the no-goby treatment (2.3 ± 6.8 %) than the high-goby treatment (−13.0 ± 5.3 %) (*T*_14_ = 3.3, *P* = 0.01). Average relative growth of creek chubs was lower than other species in the experiment (treatment means ranged from −6.7 to −18.0 %), suggesting that they were particularly susceptible to cage effects. It is not clear whether creek chubs >110 mm were more susceptible to cage effects than smaller individuals, but this would be consistent with the observed size-specific patterns amongst treatments.

### Fish stomach contents

**Fig. 2:**
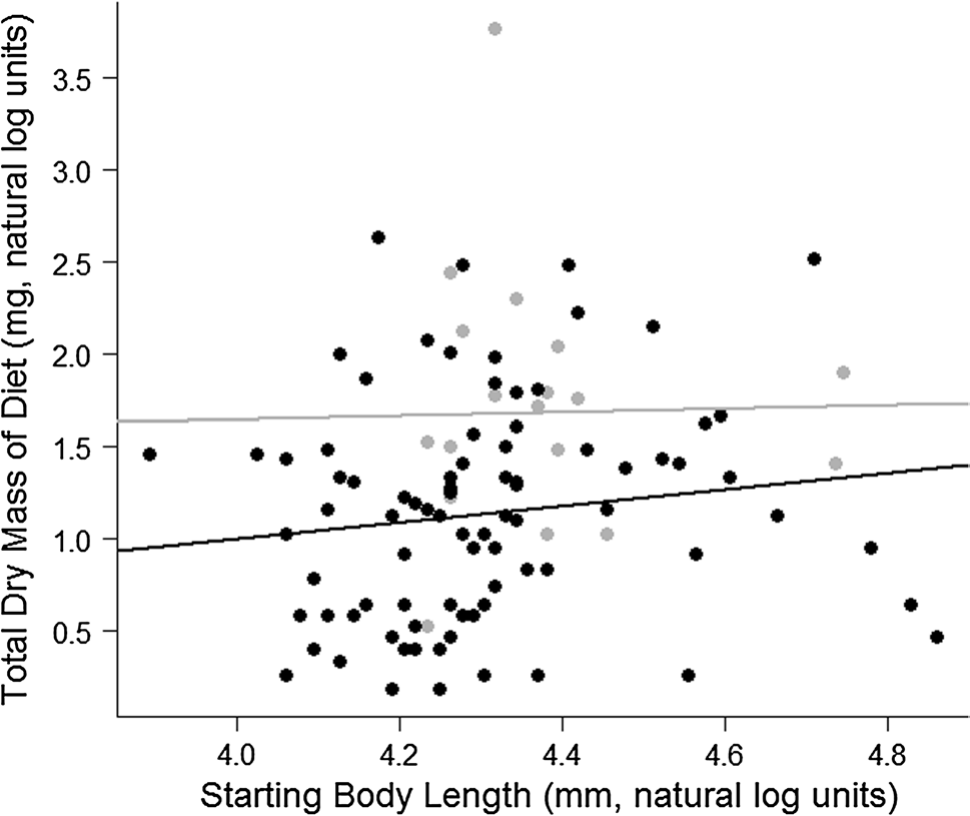
Difference in shell-free gut content weight of round goby (*Neogobius melanostomus*) between low-goby (*gray circles* and *line*) and high-goby (*black circles* and *line*) treatments. Mean gut content weight was greater in the low-goby treatment than the high-goby treatment (*F*_1,101_ = 11.2, *P* = 0.001). Data were analysed using an ANCOVA with fish length as a covariate. All diets were collected at the end of the experiment (14 September 2009, Little Suamico River, Wisconsin, USA). *n* = 18 and 87 individuals for the low and high density treatments, respectively

Significant differences in the intercepts of the relationship between gut content weight and body size (ANCOVA) were interpreted as an effect of round goby density (Fig. 2). Round goby shell-free gut content weight was 2.4-fold heavier in the low-goby treatment than the high-goby treatment on average, and significantly greater in the low-goby treatment for individuals of all sizes (*F*_1,101_ = 11.2, *P* = 0.001, Fig. 2). Conversely, gut content weight of native species did not differ among round goby density treatments (*F*_2,30_ = 1.5, *P* = 0.23 for Johnny darter; *F*_2,20_ = 1.0, *P* = 0.37 for white sucker; *F*_2,27_ = 1.6, *P* = 0.21 for creek chub) despite some diet overlap between round gobies, Johnny darters, and creek chubs (Schoener Index values = 0.54 for goby vs. darter and 0.45 for goby vs. chub).

### Fish recapture rates

Percent of fish recaptured ranged from 58 to 86 % (mean = 76 %) and total recapture rates did not significantly differ across treatments (*F*_2,13_ = 0.4, *P* = 0.66, one-way ANOVA). However, percent of recaptured individuals was significantly different among treatments for Johnny darter (*F*_2,13_ = 12.5, *P* < 0.001) and white sucker (*F*_2,13_ = 7.7, *P* = 0.006). Tukey HSD post hoc tests indicated that these patterns were driven by significantly lower recapture rates in the high-goby treatment relative to the goby-free treatment for Johnny darters (*T*_9_ = 7.5, *P* < 0.001) and white suckers (*T*_9_ = 3.5, *P* = 0.007). These patterns could be interpreted as trends in mortality.

### Benthic invertebrate biomass

Total dry mass of invertebrates, the most important fish prey, was consistent among treatments at the beginning of the experiment (*F*_2,15_ = 0.003, *P* = 0.99), indicating that no treatment group began with an advantage in prey abundance. Most stream taxa successfully colonized the enclosures after the 7 day re-colonization period, as high numbers of Chironomids, Ephemeropterans (including Caenidae, Heptageniidae, Siphlonuridae, and Leptophlebiidae), Trichopterans (including Phryganeidae and Rhyacophilidae), Coleopterans, Isopods, and Bivalves were captured (data not shown). At the end of the experiment, total dry mass of invertebrates did not differ amongst treatments or the natural stream (*F*_3,17_ = 0.15, *P* = 0.93) (ESM Fig. 2). Because fish species may have targeted certain taxa, we also compared taxon-specific dry mass for invertebrates at the end of the experiment (ESM Table 1) and found no significant treatment effects (all *P* ≥ 0.11).

## Discussion

Introduced species provide a unique opportunity to observe how intraspecific and interspecific interactions combine to shape communities because coevolved species experienced these forces in the distant past (Bruno et al. 2005). Despite this, only interspecific interactions are addressed in most instances, even though intraspecific interactions may be a key density-dependent factor. Our study illustrates how linkages between inter- and intraspecific interactions are relevant to the relationship between invasive species density and impact. At low densities, round goby relative growth was high while native fishes had reduced relative growth compared to no-goby treatments. It is worth noting that negative effects on native fish growth rates were observed at round goby densities typical of Great Lakes tributaries. Conversely, round gobies experienced reduced growth rates at high densities, indicative of increased intraspecific competition. Growth of white suckers and Johnny darters in the high-goby treatment was comparable to the no-goby treatment, suggesting weakened competition between introduced and native species. These results are consistent with studies examining competition among species in their native range, where theory and observations predict that intraspecific interactions should be stronger than interspecific interactions (Goldberg and Barton 1992; Mangla et al. 2011; Villemereuil and López-Sepulcre 2011). Although this is not universally supported by empirical studies (Gurevitch et al. 1992; Blanchet et al. 2008), this pattern may be explained by greater niche similarity between conspecifics compared to heterospecifics (Goldberg and Barton 1992; Mangla et al. 2011) or by rivalry amongst conspecifics for territory or mates (Connell 1983; Walls 1990; Macedonia and Stamps 2010).

We infer that negative effects on native fish were from interspecific interactions and not forage limitation because both total and taxon-specific invertebrate biomass were comparable amongst treatments, as was gut content weight of native fishes. This is supported by earlier work that demonstrated interference competition between round gobies and native benthic fishes (mottled sculpin and logperch) in the laboratory (Dubs and Corkum 1996; Balshine et al. 2005). In addition, Janssen and Jude (2001) proposed that interference competition, rather than resource competition, was the primary mechanism for declines in mottled sculpin populations following round goby invasion of Calumet Harbor, Lake Michigan. Our results also suggest intraspecific competition among round gobies is likely based on interference and not forage competition, as growth rates and gut content weights were reduced at high density despite equal availability of invertebrate forage. Although variation amongst individual round goby growth rates (Fig. 1) suggests that there may be some element of forage competition amongst individuals, prey availability should have been reduced at high goby densities if foraging competition was the predominant factor. Therefore, interference competition appears to be the most likely explanation for the observed patterns. This finding is supported by laboratory experiments that describe aggressive interactions between individual round gobies and a negative relationship between round goby density and per capita food consumption (Fitzsimmons et al. 2006).

Our findings support the hypothesis that the relationship between interspecific interaction strength and invasive species density follows the familiar curve of other density-dependent factors, with interspecific interactions peaking at moderate densities. This hypothesis is widely supported for interactions between indigenous species (see citations above), but contradicts the expectation that the impact of an introduced species will increase with its density (Yokomizo et al. 2009). We suggest that impact may peak at moderate invasive species densities in situations where impact is a direct result of interactions with other species. If an invader’s impact is through interspecific competition, for example, it is plausible that impact would peak at moderate densities when intraspecific competition due to resource similarity or conspecific rivalry remains low (Connell 1983; Macedonia and Stamps 2010). Furthermore, moderate invasive species densities (and peak impact) may prevail in cases where intraspecific interactions play a self-regulating role. This may explain why round gobies have been observed at lower densities in some Great Lakes tributaries than the Great Lakes themselves (Kornis et al. 2013). Density-mediated intraspecific interactions can promote coexistence amongst heterospecific competitors (Stoll and Prati 2001; Grether et al. 2009), which may partially explain why native species are usually not extirpated from invaded freshwater communities (Moyle and Light 1996).

Other potential mechanisms that could result in a hump-shaped relationship between impact and density include nutrient enrichment and cannibalism. These potential mechanisms were not explicitly tested by our experiment; however, they have been described for other species. For example, a recent laboratory study found that an invasive gastropod *Potamopyrgus antipodarum* had a negative effect on the growth rate of native gastropod *Valvata humeralis* at low densities, but had a positive effect on *V. humeralis* growth rates at high densities (Gates 2012). A subsequent field experiment found that *P. antipodarum* egested significantly more phosphorus than *V. humeralis*, suggesting nutrient enhancement as a potential mechanism for increased *V. humeralis* growth rates at high *P. antipodarum* densities (Gates 2012). In addition, cannibalism could also result in invasive species impacts peaking at moderate densities. Filial cannibalism (consuming offspring of one’s own species) is a fairly common occurrence amongst teleost fishes (Manica 2002), and cannibalism rates are often positively correlated with density (Fessehaye et al. 2006). Filial cannibalism has been observed in several invasive species including cane toad *Rhinelle marina* in Australia (Pizzatto and Shine 2011), Nile tilapia *Oreochromis niloticus* in North and South America (Fessehaye et al. 2006), rainbow smelt *Osmerus mordax* in inland lakes of eastern North America (Evans and Loftus 1987), and Nile perch *Lates niloticus* in Africa (Ogutu-Ohwayo 1990). Cannibalism has also been observed in round gobies in the Great Lakes (Yavno and Corkum 2011), and may contribute to observations of juvenile round gobies over suboptimal sandy substrate (Ray and Corkum 2001; Johnson et al. 2005). However, we believe round goby cannibalism was likely not a factor in our experiment because (1) we had no direct observations of this occurring from our analysis of gut contents and (2) the size range of round gobies used in our treatment (58–132 mm) probably precluded cannibalistic interactions.

Our results should be interpreted with care due to artificially induced levels of invasive species density and because enclosures are inherently different from the natural setting in that they restrict organism movements. Hump-shaped relationships between density and impact may be transient in nature because the requisite high densities could result in density-dependent dispersal as a mechanism to alleviate negative intraspecific interactions (Van den Bosch et al. 1992). In fact, our observation of poor growth and reduced gut content weight in round gobies from high density treatments is consistent with a density-dependent mechanism for upstream dispersal of round gobies in Great Lakes tributaries. Recent studies have documented rapid upstream expansion of round gobies in Great Lakes tributaries, ranging from 0.5 km year^−1^ (Bronnenhuber et al. 2011) to 1–4 km year^−1^ (Kornis et al. 2012). Given reports of small (5 m^2^) home ranges in the Great Lakes (Ray and Corkum 2001), such rates would be unexpected in the absence of a density-dependent dispersion response. Density-dependant dispersal has been observed in several species invasions (Hastings et al. 2005) and has been proposed as the most plausible explanation for observations of juvenile round gobies occupying suboptimal sandy substrate in areas of high round gobies density in the Great Lakes (Charlebois et al. 1997; Chotkowski and Marsden 1999; Ray and Corkum 2001; Johnson et al. 2005). Substantiating this hypothesis through direct observation in tributaries could be the focus of a future study.

In addition, although individual growth rates are often negatively correlated with density (Stiling 1988; Post et al. 1997), we did not expect a large effect for round gobies because they are regularly found at densities of 10.7 m^−2^ or greater in the Great Lakes (Corkum et al. 1998; Steinhart et al. 2004; Johnson et al. 2005). Nevertheless, round goby growth rates were much lower in the high-goby treatment than the low-goby treatment, with over 60 % of individuals experiencing negative growth. This discrepancy may be related to several differences between the Great Lakes and their tributaries that could contribute to reduced niche opportunity (sensu Shea and Chesson 2002) for round gobies in streams. First, a preferred diet item of adult round gobies, dreissenid mussels (*D. polymorpha* and *bugensis*), are abundant in the Great Lakes but typically absent from tributaries (Kornis and Vander Zanden 2010). On average, dreissenid mussels comprise about 50 % of round goby diets by mass in the Great Lakes, and round gobies must compete with a diverse suite of native benthivorous fishes for other prey (largely Chironomidae, Ephemeroptera and Trichoptera) in streams (Kornis et al. 2012). Second, invaded tributaries are typically warmer than Great Lakes sites. Warmer temperatures are associated with greater metabolic energy demands and faster growth rates (Lee and Johnson 2005); both would lead to greater food demands for stream round gobies, which could result in more frequent aggressive interactions.

It is important to note that significantly lower recapture of Johnny darters and white suckers in the high-goby treatment, if interpreted as mortality, potentially confounds the interpretation of our results. Higher escape rates from high density enclosures was unlikely because each enclosure had identical construction. Therefore, mortality seems like the probable cause of this pattern, although no carcasses were found. Fewer individuals of these species in the high-goby treatment may indicate predation (one tagged Johnny darter was found in the belly of a creek chub, but white suckers were likely too large to be susceptible) starvation (no evidence from collected diets or invertebrates), or disease (no evidence of fish disease in enclosures or in the study stream). Regardless of mechanism, reduced numbers of Johnny darter and white sucker could diminish intraspecific interactions within each species and contribute to higher individual growth rates in the high goby treatment. However, because the differences in mean densities of Johnny darters and white suckers among the treatments were so small (average of 0.7 fewer Johnny darters and 0.5 fewer white suckers per m^2^ in the high-goby treatment relative to the no-goby treatment), it is unlikely that these were responsible for the dramatic differences we observed in relative growth rates.

In summary, understanding density-impact relationships is an important goal of invasive species research, and our results illustrate how this relationship may change as the invasive species reaches densities where self-regulating factors become apparent. Although the impact of an invasive species is expected to increase with density, we found greater impacts at moderate goby densities. It remains to be seen whether such patterns are observed elsewhere, but the implications are notable given a recent study reporting that aquatic invasive species are most often found at low to moderate densities, and only rarely become highly abundant (Hansen et al. 2013). Our study suggests invasive species could have high impacts at low or moderate densities, and we caution against the assumption that impact always increases with invasive species density. Our findings invite further investigation into density-impact relationships due to their importance for understanding and managing invaded ecosystems.

## Electronic supplementary material

Below is the link to the electronic supplementary material.

Supplementary material 1 (DOCX 595 kb)
