# Supplementary material for: Experimental evidence that ecological effects of an invasive fish are reduced at high densities
Source: Oecologia. 2014 Feb 17;175(1):325–34. doi: 10.1007/s00442-014-2899-5 (PMC3992221; doi:10.1007/s00442-014-2899-5)
Supplement: Supplementary file 1 — Supplementary material 1 (DOCX 595 kb) [file 442_2014_2899_MOESM1_ESM.docx]

**Experimental evidence that ecological effects of an invasive fish are reduced at high densities**^[[1]](#footnote-1)^

Matthew S. Kornis^a,b*^, Jedchada Carlson^a^, Gabrielle Lehrer-Brey^a^, and M. Jake Vander Zanden^a^

^a^Center for Limnology, University of Wisconsin-Madison, 680 N. Park Street, Madison, WI 53706, USA.

^b^Current address: Smithsonian Environmental Research Center, 647 Contees Wharf Road, Edgewater, MD 21037, USA.

^*^ Corresponding author (e-mail: kornism@si.edu)

**ESM Appendix 1 – Description of enclosure construction.**

We used relatively large enclosures to simulate realistic fish density conditions with multiple individuals per species. This design created spatial and temporal constraints that limited our experiment to a total of three treatments. Enclosure frames were constructed from 1.9 cm diameter PVC pipe and were 1.5 x 1.5 x 0.6 m, covering an area of 2.25 m^2^. Black vinyl mesh (3.18 x 3.18 mm mesh size) was affixed to all four sides and the bottom of each frame. Mesh was overlapped and sewn together at junctions to prevent fish escape. Closed bottoms were necessary because nearly all fishes escaped from an earlier design with open-bottoms and natural substrate. Stream bottoms at each enclosure site were cleared to bare sand/gravel to facilitate an even fit with the stream bed. To simulate natural substrate conditions, fine substrate (sand/gravel) was placed inside the enclosures, followed by cobbles and boulders from an undisturbed section of stream to minimize invertebrate loss (ESM Figure 1). Enclosures were also covered with a heavy-duty vinyl mesh (5 x 10 cm openings) to prevent predation by terrestrial vertebrates while allowing light penetration and invertebrate infall.


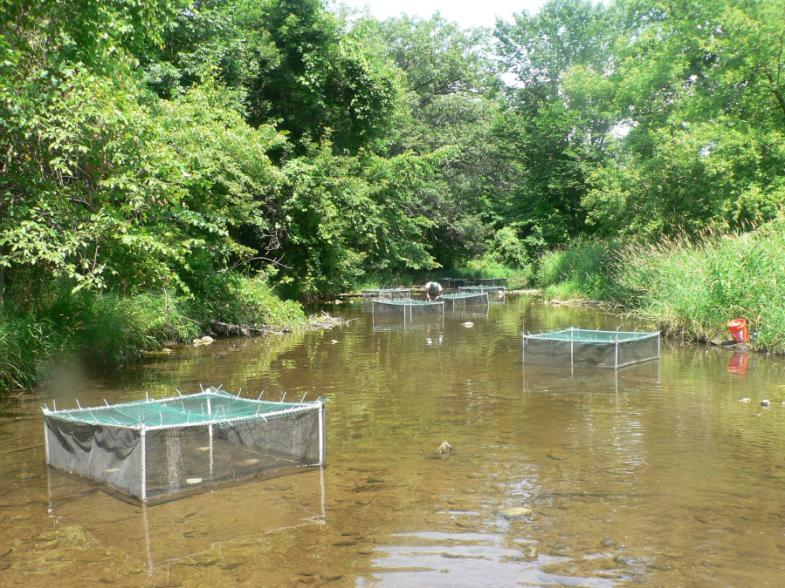

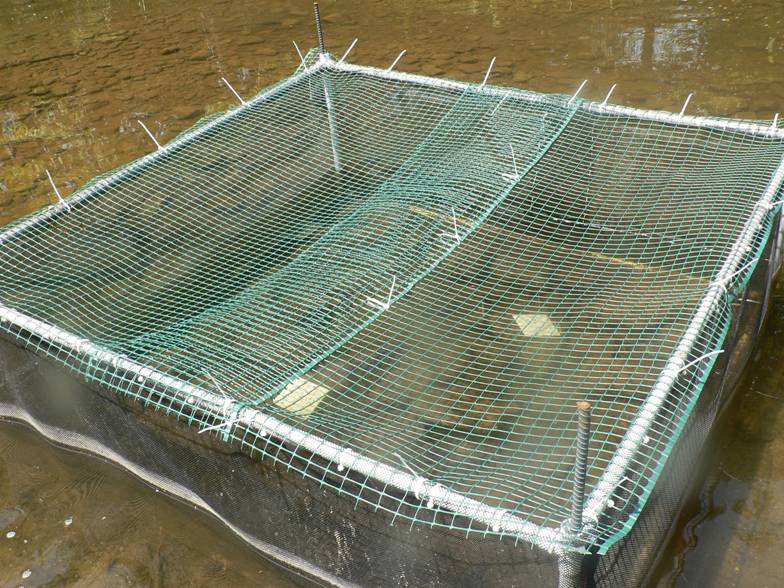

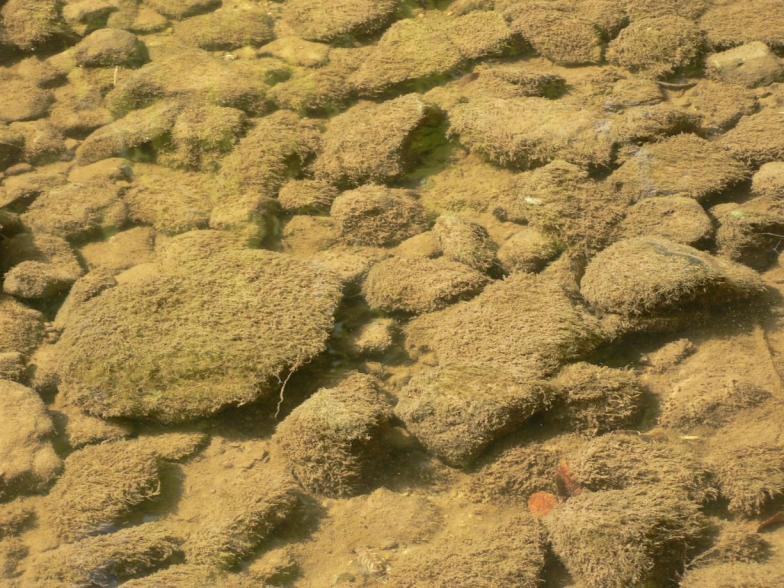

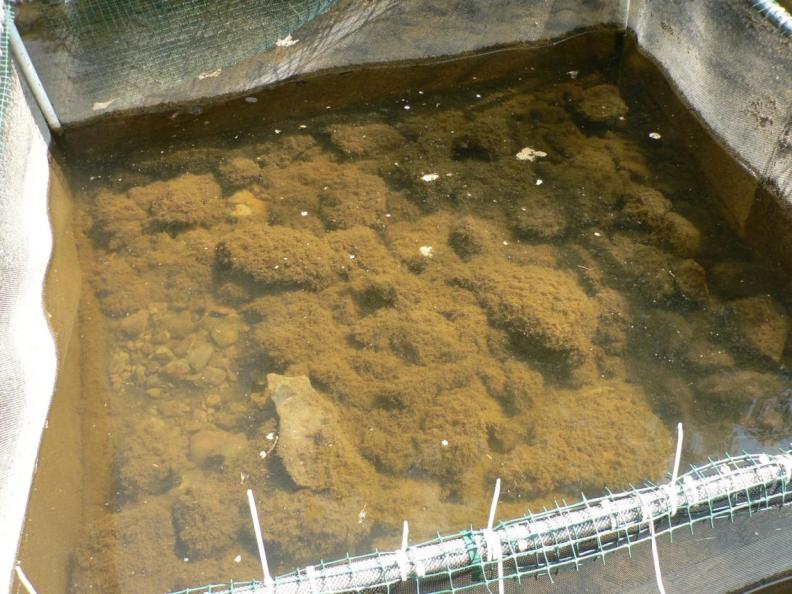

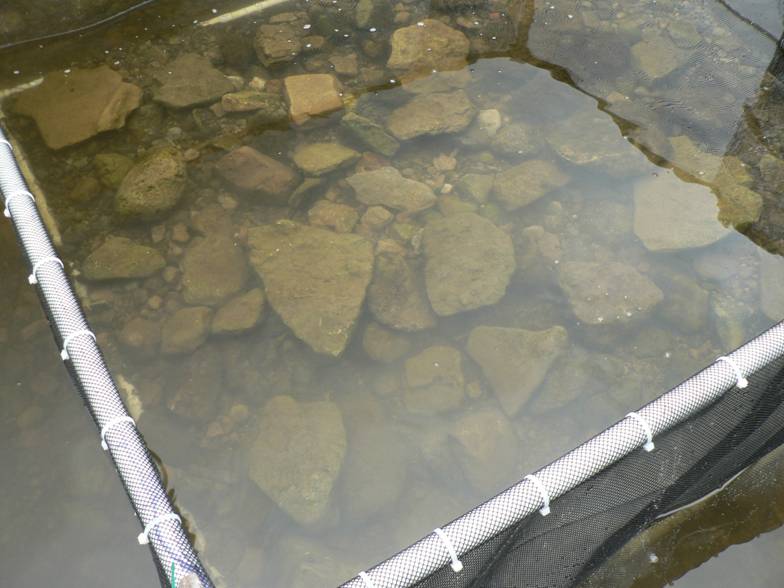

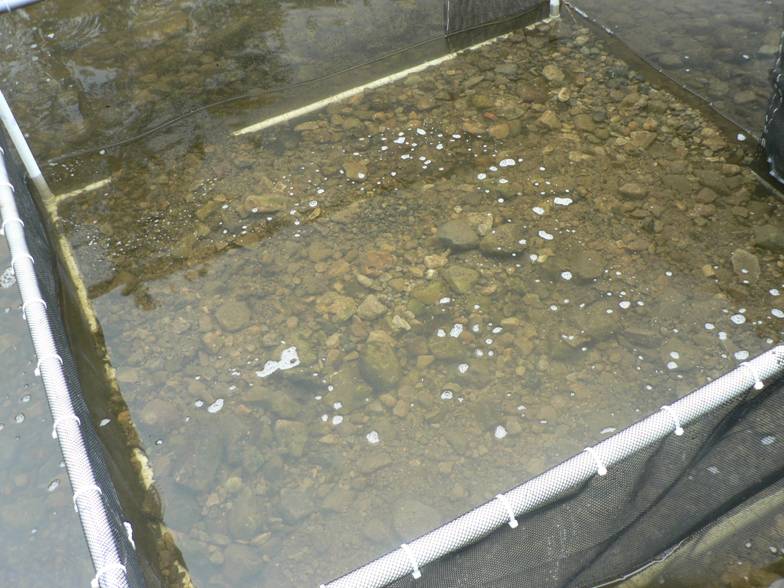


**c**

**d**

**a**

**b**

**e**

**f**

**ESM Figure 1:** Photos of experimental design. a) Small substrate (sand and gravel) was added to each enclosure to simulate the underlying substrate layer of the natural stream. b) A layer of larger substrate (cobbles and boulders) was then set on top of the small substrate base layer. Periphyton growth and substrate structure in the enclosures (c) mirrored that of the natural stream (d). e) Enclosures were covered to prevent predation while minimizing unnatural shading. f) Enclosures were placed on alternative banks at least 20 meters away from each other with a corner facing upstream to minimize drag. Enclosures were installed from 7 July 2009 through 14 September 2009 in the Little Suamico River, Wisconsin.

**ESM Appendix 2 – Macroinvertebrate data**

**
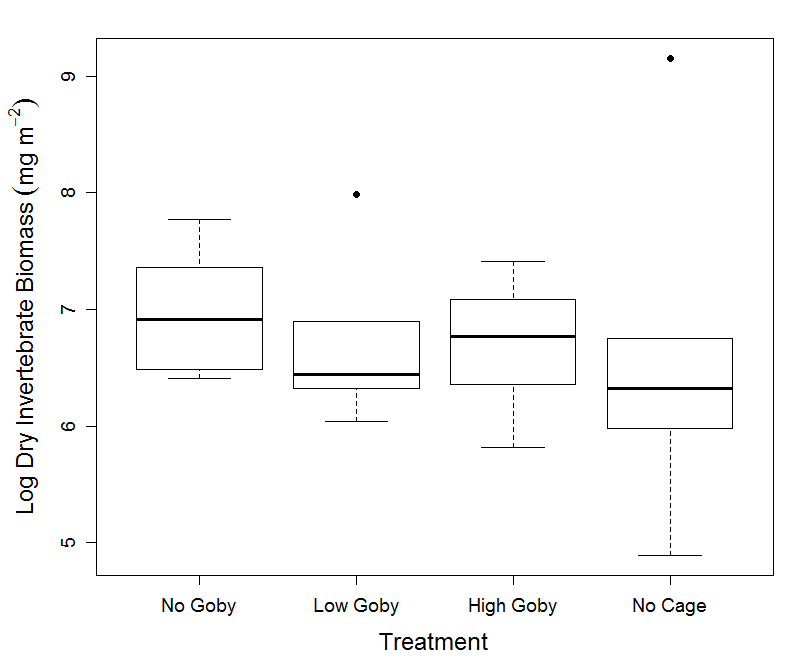
**

**ESM Figure 2**: Effects of round goby (*Neogobius melanostomus*) density on total invertebrate dry mass. In all boxes the solid black line represents the median value and the box edges the 1^st^ and 3^rd^ quartiles. Total invertebrate biomass (F_3,17_ = 0.15, *P* = 0.93) did not significantly differ among treatments. Data are from samples taken at the end of the experiment. “No cage” treatment reflects samples taken from cage-free areas of stream. n = 5, 5, 6, and 6 for number of replicates in the no goby, low goby, high goby, and no cage treatments, respectively. Enclosures were installed from 7 July 2009 through 14 September 2009 in the Little Suamico River, Wisconsin.

**ESM Table 1**: Taxon-specific differences in invertebrate dry mass (mg m^-2^) among round goby (*Neogobius melanostomus*) density treatments. No goby, low goby, and high goby treatments contain 0, 2.7, and 10.7 gobies m^-2^. Standard error is reported in parentheses. *P* values represent results of a one-factor ANOVA test with treatment as the predictor variable and taxa-specific dry mass as the response variable. “No Cage” samples were removed from analysis to test for experimental effects only. n = 5, 5, and 6 for number of replicates in the no goby, low goby, and high goby treatments, respectively. All samples were collected at the end of a 52 day experiment (14 September 2009) from enclosures installed in the Little Suamico River, Wisconsin.

| **Taxon** | **No Goby** | **Low Goby** | **High Goby** | ***P* value** |
| --- | --- | --- | --- | --- |
| Chironomidae | 208.2 (61.2) | 188.6 (104.9) | 224.0 (86.0) | 0.95 |
| Trichoptera | 55.1 (27.8) | 1.8 (1.4) | 28 (25.5) | 0.28 |
| Ephemeroptera | 358.9 (83.9) | 389.1 (87.0) | 503.6 (126.8) | 0.57 |
| Heptageniidae (Ephemeroptera) | 95.8 (23.2) | 106.6 (24.2) | 177.5 (51.9) | 0.23 |
| Siphlonuridae (Ephemeroptera) | 4.9 (1.9) | 1.2 (0.5) | 1.5 (0.8) | 0.11 |
| Caenidae (Ephemeroptera) | 137.5 (41.8) | 141.2 (26.9) | 229.6 (74.4) | 0.38 |
| Leptophlebiidae (Ephemeroptera) | 120.7 (28.2) | 140.1 (64.0) | 94.9 (26.1) | 0.76 |
| Isopoda | 134.7 (52.0) | 141.2 (49.7) | 53.1 (23.3) | 0.35 |
| Coleoptera | 71.2 (12.0) | 35.3 (11.7) | 74.4 (32.2) | 0.35 |
| Bivalvia | 110.7 (90.0) | 31.5 (27.7) | 0 (0) | 0.42 |
| Gastropoda | 53.8 (34.8) | 296.5 (214.7) | 2.5 (1.8) | 0.22 |

1. Author Contributions: MSK and MJV formulated the idea and designed the experiment. MSK performed the experiment. JC and GL collaborated in diet and invertebrate analyses. MSK performed statistical analyses. MSK and MJV wrote the manuscript; other authors provided editorial advice. [↑](#footnote-ref-1)
